# Tandem Repeat Analysis for Surveillance of Human *Salmonella* Typhimurium Infections

**DOI:** 10.3201/eid1303.060460

**Published:** 2007-03

**Authors:** Mia Torpdahl, Gitte Sørensen, Bjørn-Arne Lindstedt, Eva Møller Nielsen

**Affiliations:** * Statens Serum Institut, Copenhagen, Denmark; † Danish Veterinary and Food Research, Copenhagen, Denmark; ‡ Norwegian Institute of Public Health, Oslo, Norway

**Keywords:** *Salmonella*, Typhimurium, surveillance, outbreak, phagetyping, PFGE, VNTR, MLVA, research

## Abstract

In Denmark, as part of the national laboratory-based surveillance system of human enteric infections, all *Salmonella* Typhimurium isolates are currently subtyped by using phage typing, antimicrobial resistance profiles, and pulsed-field gel electrophoresis (PFGE). We evaluated the value of real-time typing that uses multiple-locus-number tandem-repeats analysis (MLVA) of *Salmonella enterica* serotype Typhimurium (*S*. Typhimurium) to detect possible outbreaks. Because only a few subtypes identified by PFGE and phage typing account for most infections, we included MLVA typing in the routine surveillance in a 2-year period beginning December 2003. The 1,019 typed isolates were separated into 148 PFGE types and 373 MLVA types. Several possible outbreaks were detected and confirmed. MLVA was particularly valuable for discriminating within the most common phage types. MLVA was superior to PFGE for both surveillance and outbreak investigations of *S*. Typhimurium.

## Abstract 2

Multiple-locus variable-number tandem repeats analysis improves surveillance and outbreak investigations.

Members of the bacterial genus *Salmonella* are among the major pathogens that cause infections in humans and animals. Most human *Salmonella* infections are thought to be associated with foodborne transmission from contaminated animal–derived meat and dairy products (*1*). *Salmonella enterica* subspecies *enterica* serotype Typhimurium is the second most commonly isolated serotype in Denmark (*2*) and in other industrialized countries (*3*).

Typing is an important tool for surveillance and outbreak investigations of human infections. Many demands are placed on new typing methods, including high discriminatory power so that unrelated and related isolates can be identified (*4*). The method should be easy to perform and interpret, and it should be possible to standardize, so that results can be exchanged between laboratories and be effective for local, national, and international surveillance (*4*). The many molecular typing techniques target different areas of the genome in attempts to assess genetic variability; however, the stability of such a target area has to be taken into account when considering how relevant the area is for typing (*4*). Too much variability will complicate the interpretation of the typing data in relation to epidemiologic information (*5*).

Pulsed-field gel electrophoresis (PFGE) is one of the most widely used typing methods in local, national, and international *S*. Typhimurium surveillance (*2**,**6**,**7*). Linking of PFGE data and epidemiologic information has resulted in tracing the origin of common-source outbreaks (*8**,**9*), but the method has also shown limitations within certain phage types of *S*. Typhimurium (*10**,**11*). Multiple-locus variable-number tandem-repeats analysis (MLVA), based on amplification of variable number of tandem repeat (VNTR) areas, is a promising typing method that seems to have high discriminatory power within clonal species. Three MLVA schemes have been developed for Shiga toxin–producing *Escherichia coli* O157 (STEC O157) that had either equal or improved discriminatory power when compared with PFGE (*12**–**14*). Several schemes have also been developed for *Salmonella*, including a general scheme for *S. enterica* subspecies *enterica* (*15*). This method was not equally discriminatory for all serotypes investigated, and schemes have been developed that are based on overlapping and on serotype-specific VNTR areas. One scheme was developed for *S*. Typhi (*16*) and another for *S*. Typhimurium (*17*); the latter showed high discriminatory power within *S*. Typhimurium and within the uniform phage type DT104 (*18*).

The purpose of our study was to evaluate the usefulness of MLVA in surveillance of human *S*. Typhimurium infections and detection of possible outbreaks. In Denmark, surveillance for *Salmonella* in humans, animals, and food is extensively coordinated (*2*). *S*. Typhimurium isolates from all confirmed human infections are routinely typed by using PFGE, phage typing, and antimicrobial resistance profiles. The same standardized methods are used for typing food and animal isolates; however, PFGE is used only for selected food and animal isolates. In a 2-year period, MLVA typing was included in routine surveillance, and we evaluated its discriminatory ability and usefulness in cluster detection and outbreak investigations. Comparisons with phage typing, PFGE typing, and epidemiologic information were included.

## Materials and Methods

### Isolates

In Denmark, fecal samples from patients with diarrhea are examined for bacterial pathogens at either the regional clinical laboratories or at the diagnostic laboratory at Statens Serum Institut (SSI). All *Salmonella* isolates were serotyped according to the Kaufman-White scheme (*19*), and all *S*. Typhimurium isolates were submitted to Statens Serum Institut for further characterization. In a 2-year period beginning December 2003, all confirmed *S*. Typhimurium isolates were collected weekly and further subtyped by using phage typing, antimicrobial resistance profiles, PFGE, and MLVA as part of national surveillance.

### Phenotypic Characterization

*S*. Typhimurium isolates were phage typed according to international standards (*20*) at the Danish Institute for Food and Veterinary Research (DFVF). Antimicrobial resistance profiles were generated from susceptibility to antimicrobial agents and were performed as MIC determinations. Sensititer (TREK Diagnostic Systems, LTD, West Sussex, England), a commercially prepared dehydrated panel, was used for the following antimicrobial agents: amoxicillin-clavulanic acid, ampicillin, apramycin, ceftiofur, chloramphenicol, ciprofloxacin, colistin, florphenicol, gentamicin, nalidixic acid, neomycin, streptomycin, sulfamethoxazole, tetracycline, and trimethoprim.

### PFGE Procedure

Isolates were grown overnight on blood plates, and PFGE was performed with *Xba*I by using the PulseNet USA protocol developed for *Salmonella* (*7*). The international standard *S*. Braenderup, H9812 (*21*) was used, and the gels were analyzed by using BioNumerics 4.0 (Applied Maths, Sint-Martens-Latem, Belgium). All bands with sizes between 33 kb and 1,135 kb were included in the interpretation of PFGE patterns, and isolates differing at 1 band were assigned a new PFGE type.

### MLVA Procedure

MLVA was performed by using the same primers and a modified version of the method previously described (*17*). Isolates were grown overnight on blood plates, and a small loopful of cells was placed directly into the PCR mix. One PCR reaction was performed with a multiplex kit from Qiagen (Hilden, Germany) in a total of 25 μL and included 2.50 pmol each of primers STTR3-F, STTR3-R, STTR6-F, and STTR6-R and 1.25 pmol each of primers STTR5-F, STTR5-R, STTR9-F, STTR9-R, STTR10pl-F, and STTR10pl-R. Amplification was performed with a GeneAmp9700 (Applied Biosystems, Foster City, CA, USA), starting with 15 min at 94°C, followed by 25 cycles of 30 s at 94°C, 1 min at 60°C, and 1.5 min at 72°C and ending with an extension step for 10 min at 72°C. The final products were separated with an ABI310 automated DNA sequencer (Applied Biosystems). Data collection and preprocessing were performed with GENESCAN (Applied Biosystems) and the internal size standard Geneflo-625 (CHIMERx, Milwaukee, WI, USA) for normalization. Fragment sizes for all loci were imported to BioNumerics 4.0, and allele numbers were assigned automatically for each strain by using arbitrary numbers. Unique allelic combinations were assigned a new MLVA, and all MLVA types are shown as fragment sizes (bp) in the following order: STTR9-STTR5-STTR6-STTR10-STTR3.

### Clusters and Outbreak Investigations

A cluster was defined as 5 isolates with the same MLVA type collected over a period of 4 weeks. Investigations were started if these isolates also were identical with PFGE and phage typing and included typing of food and animal isolates and interviews with patients. For confirmed outbreaks closely related PFGE types (differing at 1 band) and MLVA types (differing at 1 locus) were included in the investigations when isolated within a narrow time frame.

## Results

In total, 1,019 human *S*. Typhimurium isolates were characterized with PFGE, MLVA, and phage typing during the 2-year period. DT104, DT120, and DT12 accounted for 47.8% of all isolates; DT104 (including DT104b) was the most commonly isolated phage type. Approximately 20% of the isolates either were nontypeable (NT) or showed a phage pattern that did not correspond to a recognized phage type and was reported as phage type RDNC. Each of the remaining phage types accounted for <6% of the total number of isolates. Eighty-three isolates were assigned to phage types that were present for <1% of the total; these isolates are shown together as “others” in Table 1. PFGE typing resulted in discrimination within each phage type except DT40, for which all isolates were assigned the same PFGE type (Table 1). Within the most frequently seen phage types, many isolates were assigned to a single PFGE type, 85% of all isolates within DT12, 72% within DT104, and 40% within DT120 (Table 1). MLVA typing discriminated further, and all isolates were divided into 373 different MLVA types compared with a total of 148 PFGE types (Table 1). Discrimination within phage types was enhanced by using MLVA because <25% of the isolates within the 3 most frequent phage types were assigned to the same MLVA type (Table 1). Sixty-four PFGE types were represented by >1 isolate, 92% of these were divided into >1 MLVA type, and 53% were divided into >2 MLVA types. In total, 117 MLVA types were represented by >1 isolate; 44% of these were divided into >1 PFGE type; and 15% were divided into >2 PFGE types (data not shown).

**Table 1:** Phage type distribution for all isolates with a phage type abundance >1% of the total number of isolates*

Most common phage types	No. isolates (% of total)	No. PFGE types	No. isolates with most common PFGE type (%)	No.MLVA types	No. isolates with most common MLVA type (%)
104	173 (17.0)	11	125 (72)	84	34 (20)
120	161 (15.8)	24	65 (40)	36	40 (25)
12	153 (15.0)	14	130 (85)	47	37 (24)
193	60 (5.9)	26	21 (35)	28	20 (33)
U302	37 (3.6)	17	8 (22)	28	6 (16)
170	34 (3.3)	6	22 (65)	15	7 (21)
208	19 (1.9)	6	10 (53)	6	10 (53)
44	15 (1.5)	2	14 (93)	4	10 (67)
41	14 (1.4)	8	4 (29)	10	3 (21)
1	13 (1.3)	8	5 (38)	11	2 (15)
135	12 (1.2)	3	6 (50)	8	3 (25)
40	12 (1.2)	1	12 (100)	6	4 (33)
66	11 (1.1)	3	8 (73)	8	3 (27)
NT	116 (11.3)	36	33 (28)	49	31 (27)
RDNC	106 (10.4)	45	25 (24)	70	11 (10)
Others	83 (8.2)	–	–	–	–
All isolates	1019 (100)	148	–	373	–

*PFGE, pulsed-field gel electrophoresis; MLVA, multiple-locus variable-number tandem-repeat analysis.

**Figure 1:**
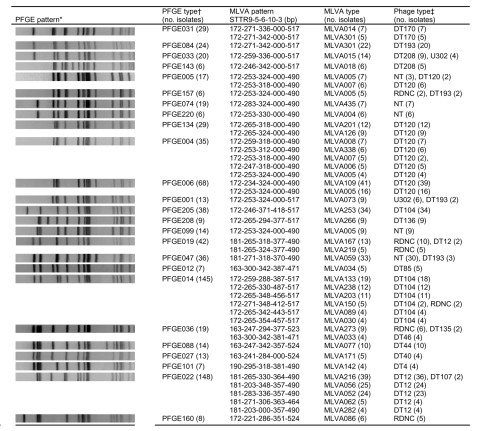
Pulsed-field gel electrophoresis (PFGE), multiple-locus variable-number tandem-repeat analysis (MLVA), phage types, and number of isolates. *PFGE patterns were sorted using the Pearson correlation in BioNumerics 4.0. †Types are shown when present 6× and when >4 isolates had identical MLVA type within each PFGE type. ‡Phage types are only shown when <2 isolates within each MLVA type had the same phage type.

Figure 1 shows the most common PFGE profiles, representing 75% of the isolates, as well as the most common MLVA and phage types within each PFGE type. Isolates within the most widespread PFGE types were separated into several MLVA types (PFGE014 and PFGE022, Figure 1), whereas isolates within more rare PFGE types often had the same or 1 frequently seen MLVA type. Isolates within PFGE types and MLVA types often had the same or closely related phage types, and isolates within each phage type had closely related PFGE and MLVA types (except NT, RDNC, and DT193). MLVA types that had the same PFGE type were mostly conserved at MLVA loci STTR3 and STTR9, whereas the other 3 loci were more variable. The plasmidborne STTR10 was missing within DT120, DT170, DT208, and U302 and present within most other phage types. Exceptions were DT193, NT, and RDNC isolates, in which STTR10 could be either absent or present (Figure 1). Other trends were observed that correlate MLVA to both PFGE and phage type, including the more stable loci STTR3 and STTR9 (Figure 1), but MLVA cannot be used to predict either the phage type or the PFGE type.

**Figure 2:**
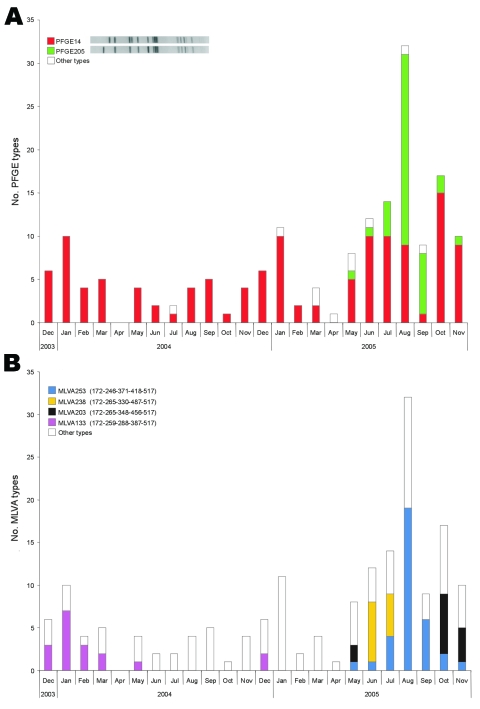
Monthly occurrence of pulsed-field gel electrophoresis (PFGE) types (A) and multiple-locus variable-number tandem-repeat analysis (MLVA) ypes (B) within Salmonella Typhimurium isolates with phage type DT104 over the 2-year study period. All PFGE and MLVA types that occurred <4× were included in other types.

Figure 2 shows the monthly occurrence of PFGE types (Figure 2A) and MLVA types (Figure 2B) within DT104. Most DT104 isolates were assigned to the same PFGE type (PFGE014) until a new PFGE type appeared in the summer of 2005 (Figure 2A). Most isolates that were assigned to this new PFGE type (PFGE205) also had a new and unique MLVA profile (MLVA253) (Figure 2B). Analyzing some isolates from animal and food products that had the same phage type and antibiotic resistance profile showed an isolate from imported beef with the same PFGE and MLVA type. An isolate with the same MLVA type was also found in Norway; this isolate originated from a patient who had been in Denmark. The rest of the isolates with the most common PFGE type (PFGE014) were divided into 83 different MLVA types (partly shown in Figure 1). Approximately 80% of the DT104 isolates were multidrug resistant (MR DT104), i.e., resistant to at least 5 microbial agents, including ampicillin, chloramphenicol, streptomycin, sulfonamides, and tetracycline. During December 2003 to March 2004, routine resistance typing detected a small cluster of isolates (cluster 1, Table 2) that diverged from the common MR DT104 (Table 2). These isolates were resistant to only ampicillin and sulfamethoxazole; when typed with MLVA, they clustered with a unique profile (MLVA133), whereas the isolates had the most common PFGE profile (PFGE014) (Figure 1). During October and November 2005, another small cluster (cluster 13, Table 2) that was not detected with PFGE was detected with MLVA typing (MLVA003). These isolates also diverged from the most common DT104 resistance pattern as they were sensitive to all antimicrobial agents.

**Table 2:** Clusters identified by MLVA typing that were investigated in the 2-y period, by typing of food and animal isolates and/or patient interviews

Cluster no. and period	No. Danish isolates (no. Norwegian isolates)	Phage type/ PFGE type/ MLVA type	Resistance profile	Description of confirmed outbreaks
1. Dec 03–Mar 04	16	DT104/PFGE014/MLVA133	Ampiclilin, sulfomethoxazole	Human cases from narrow geographic area. Isolate match from local slaughterhouse.
2. Jun–Jul 04	21 (1)	DT12/PFGE022/MLVA052	Sensitive	Human cases from narrow geographic area. Interviews indicated source from local butcher.
3. Aug–Sep 04	25 (1)	DT12/PFGE022/MLVA056	Sensitive	Human cases from narrow geographic area.
4. Aug–Sep 04	28	NT/PFGE047/MLVA059	Ampicillin, streptomycin, tetracyline	Isolate match from slaughterhouse.
5. Oct–Dec 04	9	NT/PFGE099/MLVA005	Ampicillin, sulfomethoxazole, streptomycin tetracycline	Isolate match from imported meat.
6. Jan–Nov 2005	40	DT120/PFGE006/MLVA109	Sensitive	
7. Apr–Aug 2005	15	RDNC/PFGE019/MLVA219, MLVA167	Sensitive	MLVA167 isolate match from pig herd and both MLVA167 and MLVA219 were isolated from meat from the same slaughterhouse.
8. May–Aug 2005	26	DT12/PFGE022/MLVA216	Sensitive	Human cases from narrow geographic area. Isolate match from local slaughterhouse and from local pig herd.
9. Jun–Jul 2005	12	DT104/PFGE014/MLVA238	MR	
10. Jun–Oct 2005	30 (1)	DT104/PFGE205, PFGE215/MLVA253, MLVA350, MLVA351, MLVA352	MR	Interviews indicated restaurant outbreak. Isolate match from imported beef served as carpaccio in restaurant.
11. Jul–Aug 2005	9	DT136/PFGE208/MLVA266	Sensitive	
12. Oct–Nov 2005	22	DT193/PFGE084/MLVA301	Ampicillin, sulfomethoxazole, streptomycin, tetracycline	Human cases from narrow geographic area. Interviews indicated local butcher, and several samples collected from butcher were positive for outbreak profile.
13. Oct–Nov 2005	11	DT104/PFGE014/MLVA203	Sensitive	
14. Oct–Nov 2005	7	NT/PFGE074/MLVA435	Ampicillin, sulfomethoxazole, streptomycin, tetracycline	

*MLVA, multiple-locus variable-number tandem repeat analysis; PFGE, pulsed-field gel electrophoresis.

**Figure 3:**
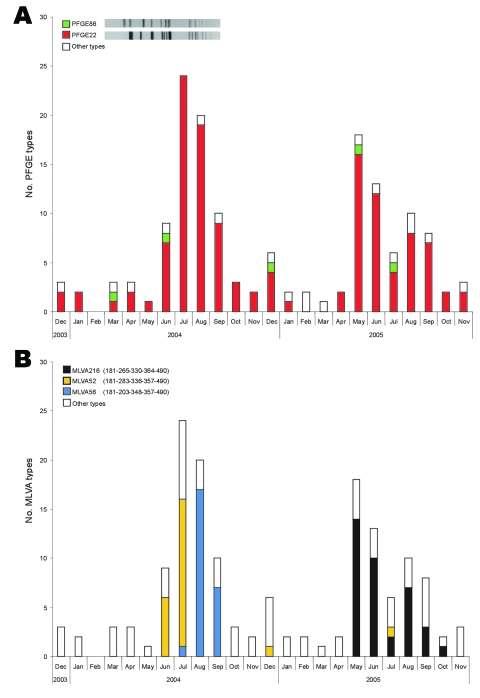
Monthly occurrence of pulsed-field gel electrophoresis (PFGE) types (A) and multiple-locus variable-number tandem-repeat analysis (MLVA) types (B) within Salmonella Typhimurium isolates with phage type DT12 over the 2-year study period. All PFGE and MLVA types that occurred <4× were included in other types.

Figure 3 shows the monthly occurrence of PFGE types (Figure 3A) and MLVA types (Figure 3B) within DT12. A high fluctuation was seen for DT12, but no clusters were detected from PFGE typing because 85% of all the isolates had the same PFGE type (PFGE022) (Table 1 and Figure 3A). MLVA typing showed 3 major clusters over the 2-year period, 1 in June and July 2004 (MLVA052), 1 in August and September 2004 (MLVA056), and 1 from May to October 2005 (MLVA216) (Figure 3B). The last cluster (cluster 8, Table 2) was confined to 1 region in Denmark; 1 isolate from a local slaughterhouse was positive for this type by MLVA typing of a wide selection of animal and food DT12 isolates. A national outbreak was indicated by PFGE typing and geographic distribution of the higher incidence of PFGE022 isolates in the summer of 2004 (Figure 3A). MLVA typing separated the cluster into 2 major types (Figure 3B). From a comparison of MLVA type, geographic area, and date of isolation, it was concluded that what was originally thought to be 1 outbreak was actually caused by 2 different MLVA types (MLVA052 and MLVA056), which differed at loci STTR5 and STTR6 with 13 and 2 repeat units, respectively (Figure 1). One outbreak was confined to a county in Jutland in June and July (cluster 2, Table 2), and the other was confined to the Copenhagen area in August and September (cluster 3, Table 2). The first cluster was also epidemiologically linked to a specific butcher shop, whereas no apparent source was found for the latter cluster. Two Norwegian patients who had been traveling to Denmark were identified. Characterization of these isolates showed that 1 patient was infected with MLVA52 and the other with MLVA56.

## Discussion

Further investigations into clusters are started in Denmark when >5 *S*. Typhimurium isolates have the same type and are isolated within a 4-week period. In total, 14 clusters and possible outbreaks were detected and further investigated over the 2-year period (Table 2). For more than half of these clusters, a likely common source was found either by typing of veterinary and food isolates or by patient interviews (Table 2). Seven of these clusters would not have been detected when only using PFGE typing because isolates had the most common PFGE type within the assigned phage type. Two clusters would have been further divided if clusters were only assigned from MLVA types (Table 2). Cluster 7 contained 2 MLVA profiles that differed from each other with 1 repeat unit at STTR6 (MLVA167 and MLVA219 in Figure 1), but all isolates had a unique RDNC phage type that had not been identified before in Denmark, and both MLVA types were isolated from meat from the same slaughterhouse (Table 2). Cluster 10 contained 4 different MLVA profiles; most isolates had the same MLVA type, but 3 isolates were assigned to MLVA types that differed at STTR6 with 1, 2, or 8 repeat units, respectively. One isolate was also included in cluster 10 that differed at 1 band to the most common PFGE profile but had the most common MLVA type. Clusters that could be detected with MLVA were often supported by a unique PFGE profile (clusters 4, 10, 11, 12 and 14; Table 2), a characteristic antimicrobial resistance profile that differed from what was normally seen within the concerned phage types (clusters 1, 4, 12, 13, and 14; Table 2), or epidemiologic information, or typing of food and animal isolates (clusters 1, 2, 3, 4, 5, 7, 8, 10, and 12; Table 2)

In Denmark, *S*. Typhimurium accounts for ≈30% of all human *Salmonella* infections (*2*), and as part of the national surveillance, all human *S*. Typhimurium isolates are subtyped by using phage typing, PFGE typing, and antimicrobial resistance profiles. These typing methods are not always discriminatory enough for surveillance and detection of common-source outbreaks. Some of the most common PFGE types account for a high percentage of isolates within each phage type and are also among the most common PFGE types in other European countries (*22*). We started routine MLVA typing (*17*) of all *S*. Typhimurium isolates over a 2-year period.

Our data supported the improved discrimination of MLVA within the uniform phage type DT104 (*18*) and furthermore showed an enhanced discrimination when compared with PFGE for almost all other phage types investigated (Table 1). The improved discrimination when we used MLVA was dependent on the phage type investigated, but with the *S*. Typhimurium level that is seen in Denmark, MLVA was especially useful for detecting clusters in the most common phage types, DT104, DT120, and DT12 (47.8% of all *S*. Typhimurium isolates), as well as isolates assigned to either NT or RDNC (21.7% of all *S*. Typhimurium isolates). No other phage types accounted for >6% of the total number of isolates, and PFGE typing would probably be sufficient for cluster detection within these less common phage types. In rare phage types, present in 1% of the total number of isolates, most of the isolates had the same antimicrobial resistance profile, PFGE type, and MLVA type; therefore, phage typing would probably be sufficient for detecting possible human outbreaks. On the other hand, phage typing would probably not be sufficient when trying to trace the source to animal or food isolates because phage types that are rare in humans can be common in animals and food (e.g., DT170 and DT193).

Most clusters that were detected with MLVA were supported by a unique phage type, PFGE profile, or antimicrobial resistance profile but none of these methods would have resulted in detecting as many clusters if used alone for surveillance. Both MLVA and PFGE were variable within clusters that were defined by other typing methods. One cluster defined by both PFGE and MLVA included 2 PFGE types that differed in 1 band and 4 MLVA types that differed at 1 locus. Another cluster contained 2 MLVA types, but all isolates had the same RDNC profile and PFGE type, which indicated that these patients were infected by a common source. For STEC O157, including isolates that differ by 1 repeat unit at 1 or 2 loci in outbreak investigation has been suggested (*13**,**14*). Results from our study suggest that including *S*. Typhimurium isolates that differ at 1 locus but with a variable number of repeat units would be useful. If including isolates that differ at 1 MLVA locus together with date of isolation for cluster detection, all reported clusters from Table 2 would have been detected and no additional cases included. Another possibility could be to include another typing method such as phage typing or PFGE together with MLVA for surveillance and outbreak investigations.

During the 2-year study period, clusters detected with MLVA were linked to a common source by MLVA typing of animal and food isolates or with interview information. Seven clusters were linked with animal or food isolates with the same MLVA and PFGE profile. One outbreak was caused by imported carpaccio, a finding further supported by interviews that showed that most patients had eaten at the same restaurant, which served carpaccio. Another outbreak was caused by meat from a local slaughterhouse from the same region as most of the patients (*23*). Another local outbreak was caused by a local butcher; samples were found positive for the same MLVA and PFGE profile. Finally, 4 outbreaks were linked to slaughterhouses or to imported meat, samples from which were positive for the same MLVA and PFGE profile as the outbreak profile. We were unable to identify a possible source for some of the MLVA clusters, but many clusters were supported by epidemiologic information that indicated a common source. Most MLVA clusters with human cases that were linked to animal or food isolates were also supported by epidemiologic information.

For daily surveillance, MLVA has many advantages when compared with PFGE. Expensive equipment is needed to perform both processes; however, reagents for MLVA typing are cheaper and the process is less labor-intensive and faster to perform than PFGE. MLVA can be completely automated and its data are easier to analyze and interpret. The standardization of MLVA makes it possible to exchange data between laboratories. We routinely exchange data, either as fragment sizes or allelic combinations, between Denmark and Norway (*24*). Three isolates have been found in Norway that had the same MLVA profile as 3 different clusters detected in Denmark. All 3 Norwegian patients had been traveling to Denmark, and interviews revealed that 1 patient had eaten at the same restaurant as all other patients with the same MLVA type found in Denmark. MLVA has also been used to trace a common-source outbreak in Norway caused by imported meat. Two Danish patients were found with this MLVA type (*25*), and patient information showed that both patients had traveled to the same country from which the meat was imported.

In conclusion, MLVA improved surveillance of human *S*. Typhimurium infections in Denmark. MLVA was faster to perform, easier to interpret and analyze, and more discriminatory than PFGE. Several possible outbreaks were detected that otherwise would not have been detected. Some of these outbreaks were solved either by linking MLVA and epidemiologic information or by MLVA typing of animal and food isolates. We were also able to link human cases from Denmark and Norway to the same common-source outbreak. MLVA might provide an advantage to local, national, and international surveillance of *S*. Typhimurium infections.
